# Intratympanic steroid therapy for Bell’s palsy with poor prognostic results

**DOI:** 10.1038/s41598-021-87551-x

**Published:** 2021-04-13

**Authors:** Akira Inagaki, Sachiyo Katsumi, Shinji Sekiya, Shingo Murakami

**Affiliations:** Departments of Otolaryngology, Head and Neck Surgery, Nagoya City University, Graduate School of Medical Sciences and Medical School, 1 Kawasumi, Mizuho-cho, Mizuho-ku, Nagoya, Aichi 467-8601 Japan

**Keywords:** Neurology, Neurological disorders

## Abstract

In Bell’s palsy, electrodiagnosis by electroneurography (ENoG) is widely used to predict a patient’s prognosis. The therapeutic options for patients with poor prognostic results remain controversial. Here, we investigated whether early intervention with intratympanic steroid therapy (ITST) is an effective treatment for Bell’s palsy patients with poor electrodiagnostic test results (≤ 10% electroneurography value). Patients in the concurrent ITST group (*n* = 8) received the standard systemic dose of prednisolone (410 mg total) and intratympanic dexamethasone (16.5 mg total) and those in the control group (*n* = 21) received systemic prednisolone at the standard dose or higher (average dose, 605 ± 27 mg). A year after onset, the recovery rate was higher in the ITST group than in the control group (88% vs 43%, P = 0.044). The average House-Brackmann grade was better in the concurrent ITST group (1.13 ± 0.13 vs 1.71 ± 0.16, P = 0.035). Concurrent ITST improves the facial nerve outcome in patients with poor electroneurography test results, regardless of whether equivalent or lower glucocorticoid doses were administered. This may be ascribed to a neuroprotective effect of ITST due to a higher dose of steroid reaching the lesion due to dexamethasone transfer in the facial nerve.

## Introduction

Bell’s palsy is a peripheral facial nerve palsy of unknown etiology. It is the most common cause of acute peripheral facial palsy, accounting for approximately 65% of acute facial palsies^1^, with an annual incidence of 20–30 per 100,000 population^2^. Steroid treatment starting at an equivalent prednisolone dose of 30–60 mg/day is generally effective for facial nerve palsy that is moderately severe at worst, such that 71–96% of patients with Bell’s palsy are reported to have a favorable outcome^3–5^. However, this standard-of-care can be inadequate for patients with high-grade facial palsy, and indeed, many patients in this group suffer sequelae^6,7^.

The prognosis in patients with Bell’s palsy is most reliably predicted by the severity of degeneration of the facial nerve^8^. In the early phase, it is widely accepted that this severity is most accurately evaluated by electroneurography (ENoG)^9^, a technique originally developed by Esslen in 1977^10^, which compares the maximum amplitude of the compound action potential of muscles innervated by the facial nerve to electrical stimulation on the ipsilateral and contralateral sides by stimulating the facial nerves at the stylomastoid foramen. In Bell’s palsy, the intratemporal part of the facial nerve is initially affected when Wallerian degeneration is initiated, reaching the stylomastoid foramen at around 72 h. This degeneration extends toward the periphery and is complete 21 days after onset^9^. Because of this facial nerve pathology, ENoG is considered to be helpful for predicting recovery only within a time window of 3–21 days^9^.

To date, it is widely accepted that more than 90% facial degeneration on ENoG is highly correlated with incomplete recovery in Bell’s palsy^8,11–16^. While relatively few Bell’s palsy patients meet this criterion^13,15,17,18^, for patients above this critical limit, the standard-of-care is mostly insufficient, meaning the chances of complete recovery are only 0–25%^11,15,16,19–21^. A consensus on the therapeutic management for this subset of patients remains elusive, and effective and readily applicable therapeutic alternatives have yet to be developed.

Recently, we reported that early intervention with intratympanic steroid therapy (ITST) concurrently with standard therapy improves recovery in patients with Bell’s palsy^22^; however, the efficacy of ITST on patients with a poor electrophysiological result has not been determined. Therefore, we investigated whether ITST is an effective therapeutic option to improve recovery for this subset of Bell’s palsy patients.

### Statistical methods

All data are presented as the mean ± standard error of the mean unless otherwise stated. Differences between the concurrent ITST and historical control groups were assessed for statistical significance using Student’s *t*-test. Nonparametric analysis was performed for data that were not distributed normally using the Mann–Whitney rank sum test (SigmaPlot, Systat Inc, San Jose, CA). Fisher’s exact test and the chi-square test were used to analyze the statistical significance of differences in recovery rates (SigmaPlot). Further details concerning data collection and statistical analyses are provided in the Supplemental Methods.

## Results

### Participants

Forty-one patients (26 men, 15 women; mean age 45.5 ± 2.2 [24–69] years) with Bell’s palsy were identified to receive concuTrrent ITST. Eight of these patients (5 men, 3 women; mean age 40.0 ± 4.4 [26–65] years) who met the study eligibility criteria, including a ≥ 90% facial nerve degeneration on ENoG, were enrolled in the concurrent ITST group (Table 2, Supplementary Fig. 1). Medical records of 541 patients with an initial diagnosis of Bell’s palsy were reviewed to identify those eligible for the historical control group. Twenty-one of these patients (8 men, 13 women; mean age 48.2 ± 3.6 [20–73] years) met the study eligibility criteria (Table 2, Supplementary Fig. 1). No patient in the concurrent ITST group suffered from severe injection-associated complications such as residual tympanic membrane perforation or significant hearing deterioration.

### Efficacy of concurrent ITST in Bell’s palsy with ≥ 90% degeneration on ENoG

**Figure 1:**
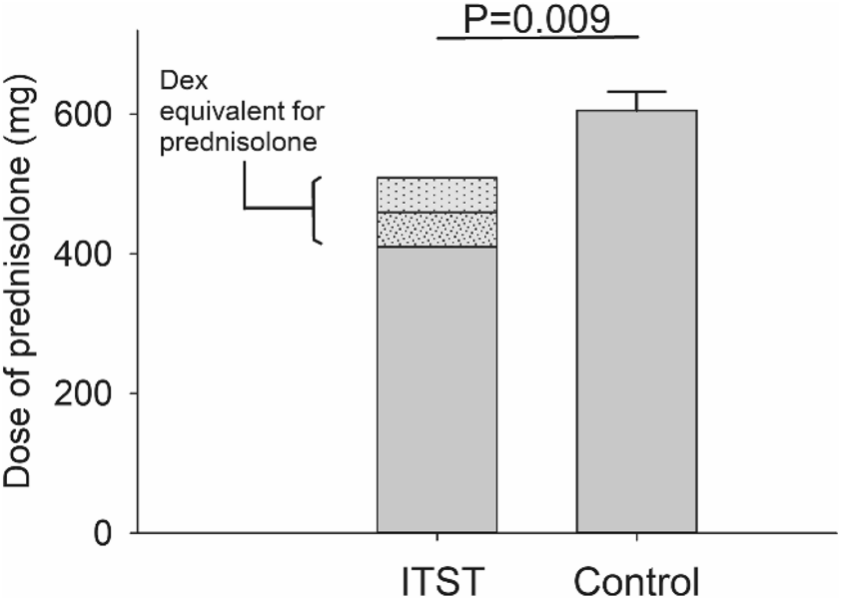
Total amount of prednisolone administered in each group. Dexamethasone 16.5 mg is assumed to be equivalent to 50–100 mg of prednisolone (see “Results”). The equivalent dose was thus regarded as 492.5 mg for the comparison between groups (unpaired *t*-test). *Dex*, dexamethasone; *ITST*, intratympanic steroid therapy.

There was no statistically significant between-group difference in the number of patients with the most severe HB grades initially (difference in mean HB grade: 0.06, P = 0.67). The concurrent ITST group received a fixed systemic steroid dose of 410 mg in addition to 16.5 mg of intratympanic dexamethasone (Table 1), while the control group received a mean prednisolone dose of 605 ± 27 mg. The glucocorticoid activity of dexamethasone is reportedly 3–6 times that of prednisolone^23,24^. Patients in the concurrent ITST group were treated with 410 mg of oral prednisolone, so the total glucocorticoid activity was likely equivalent to 460–510 mg of prednisolone. These numbers are significantly lower than the average prednisolone doses in the control group (P = 0.009). The glucocorticoid activity of concurrent ITST is assumed to be equivalent to 495.5 mg of prednisolone (Fig. 1).

**Table 1:** Standard oral steroid and antiviral therapies and concurrent ITST dexamethasone dose.

Day	Prednisolone dose, mg	Valaciclovir dose, mg	Concurrent ITST dexamethasone dose, mg (number of cases)		
			40	0	1
1	60	1000	1.65		
2	60	1000	1.65	1.65	
3	60	1000	1.65	1.65	1.65
4	60	1000	1.65	1.65	1.65
5	60	1000	1.65	1.65	1.65
6	30		1.65	1.65	1.65
7	30		1.65	1.65	1.65
8	30		1.65	1.65	1.65
9	10		1.65	1.65	1.65
10	10		1.65	1.65	1.65
11				1.65	1.65
12					1.65

Doses of prednisolone and valaciclovir specified in the standard protocols for treating severe Bell’s palsy. In this study, intratympanic steroid therapy was initiated on day 1, 2, or 3 after onset of facial palsy. Top: numbers of patients who started concurrent ITST on each of day 1, 2, and 3. ITST, intratympanic steroid therapy.

**Figure 2:**
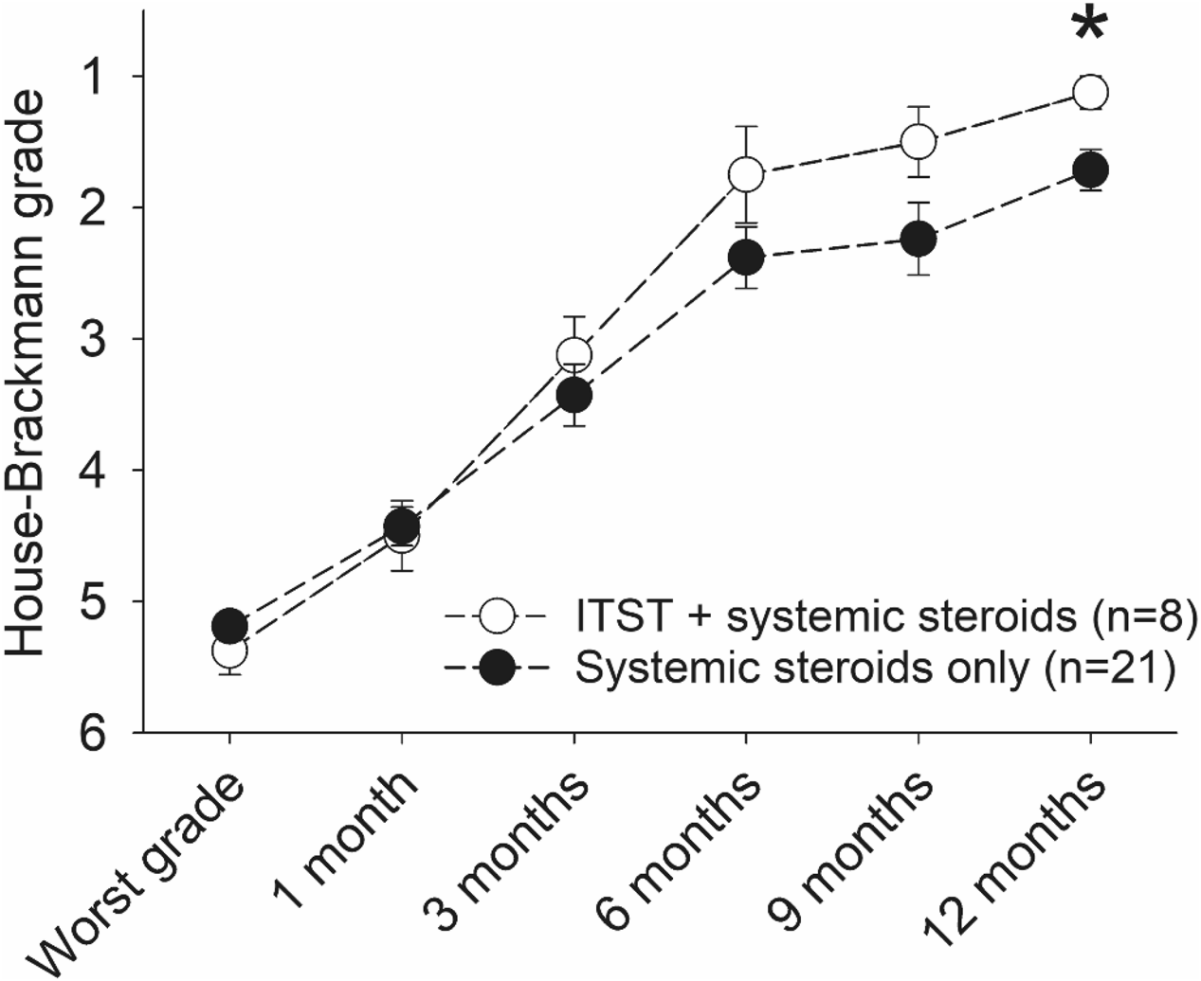
Recovery of facial nerve function in Bell’s palsy with poor electroneurography test results. The mean House-Brackmann (HB) scores for the concurrent ITST group (filled circles) and systemic steroid therapy alone group (open circles) are plotted over time. All patients exhibited poor electrophysiological test results (≤ 10% via electroneurography). *P < 0.01. *ITST*, intratympanic steroid therapy.

Although both study groups showed recovery, the concurrent ITST group recovered more rapidly (Fig. 2). The between-group difference became significant 12 months after onset (concurrent ITST group vs control group: 1.13 ± 0.13 vs 1.71 ± 0.16, P = 0.035, Table 2, Supplementary Table 2). This significant difference in recovery was also confirmed by assessments using the Yanagihara grading system (Supplementary Fig. 2). The rate of recovery to HB grade I was significantly higher in the concurrent ITST group (88%, 7/8) than in the control group (43%, 9/21, P = 0.044, Fisher’s exact test).

**Table 2:** Baseline characteristics and outcomes in patients with Bell’s palsy.

	Patients with poor electrophysiological results^c^		
	ITST (n = 8) (range)	Systemic steroid therapy (n = 21) (range)	*P*-value
Age (years)	40.0 ± 4.4 (26–65)	48.2 ± 3.6 (20–73)	0.21
Time after onset of first systemic steroid therapy (days)	2.13 ± 0.44 (0–4)	2.19 ± 0.49 (0–7)	0.67
Most severe grade	5.38 ± 0.18 (5–6)	5.19 ± 0.11 (5–6)	0.49
HB grade at 12 months	1.13 ± 0.13 (1–2)	1.74 ± 0.16 (1–3)	0.035*
Total dose of systemic prednisolone (mg)	410 (fixed) Dex 16.5 mg^a^	605 ± 27 (410–890)	0.009**
Recovery to HB grade I	7/8 (88%)	9/21 (43%)	0.044* ^b^

Dex, dexamethasone; HB, House-Brackmann; ITST, intratympanic steroid therapy.

*P < 0.05; **P < 0.01.

^a^Equivalent dosage is postulated to be 492.5 mg (see “Discussion” section).

^b^Fisher’s exact test.

^c^Based on electrophysiological test results of ≤ 10% on electroneurography.

### Correlation between facial nerve recovery and severity of degeneration

ENoG values change over time after the onset of facial palsy. This temporal change of ENoG varies between patients depending on the severity of intratemporal facial nerve pathology and the degree of Wallerian degeneration. Facial palsy with severe facial nerve damage demonstrates a rapid decline in response to electrical stimuli.

**Figure 3:**
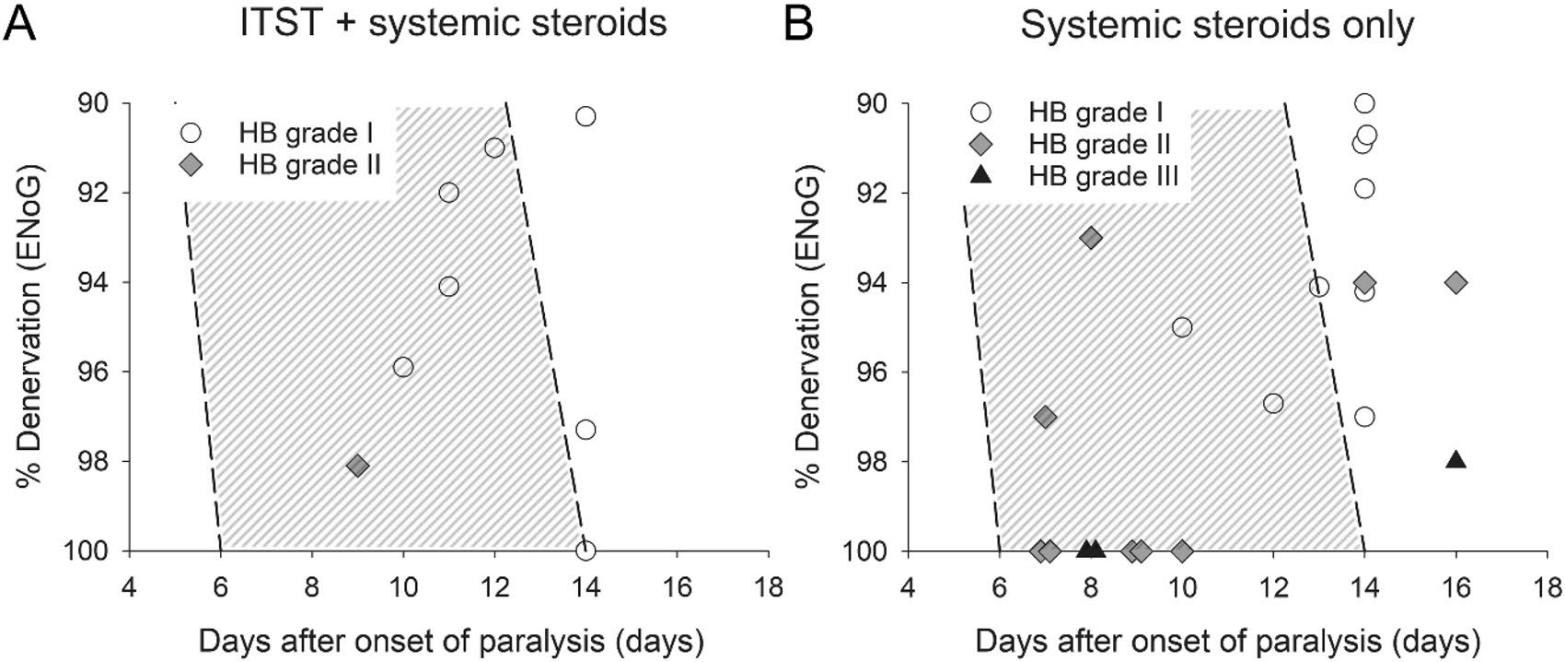
Correlation between the degree of facial nerve degeneration and days after facial palsy onset. Each symbol represents one patient’s facial outcome 12 months after facial palsy onset. The x-axis represents days after onset of facial palsy and the y-axis represents the degree of facial nerve degeneration as observed via ENoG. The shaded regions in each figure indicate the values that Fisch suggested would indicate complete degeneration of the facial nerve in Bell’s palsy^8^. Note that 6 of 8 patients in the concurrent ITST group and 11 of 21 patients in the control group had complete facial nerve degeneration according to these criteria. *ENoG*, electroneurography; *HB*, House-Brackmann; *ITST*, intratympanic steroid therapy.

Based on the chronology of ENoG values indicating complete facial nerve degeneration reported by Fisch^8^ which is widely accepted to date^21,25,26^, we investigated further the effect of concurrent ITST on facial outcome in cases with complete degeneration. This analysis identified 6 of 8 cases in the concurrent ITST group and 11 of 21 cases in the control group as cases of complete degeneration (Fig. 3A,B). Among patients with complete degeneration, 83% (5 of 6 cases) in the concurrent ITST group and 18% (2 of 11 cases) in the control group recovered to HB grade I, suggesting significantly better recovery in the concurrent ITST group (P = 0.035, Fisher’s exact test). On the other hand, 2 of 8 patients in the concurrent ITST group and 10 of 21 cases were categorized as incomplete degeneration, but demonstrated ≥ 90% facial nerve degeneration by 16 days after onset. In this category 2 of 2 (100%) patients in the ITST group recovered, which is also better than in the control group, in which 7 of 10 (70%) recovered to HB grade I; however, those two groups did not differ statistically (P = 1.0).

## Discussion

To date, the etiology of Bell’s palsy has not been identified^2^. Histological analysis reveals various microscopic findings such as inflammation with cellular infiltration^27^, and several etiological speculations, such as ischemia or reactivation of HSV-1 have been offered^25,28^. While Bell’s palsy may comprise various etiologies, severe Bell’s palsy causes irreversible histological changes in the facial nerve, which hamper functional recovery^29^. In the present study, patients in both concurrent ITST and control groups lost responsiveness to electrical stimuli; both groups presented ≥ 90% facial nerve degeneration on ENoG, but recovered differently, indicating that there was a difference in the severity of facial nerve damage between these two groups. According to the current concept of peripheral nerve injury, damage to the endoneurium of peripheral nerves, along with that of the axon and myelin sheath, i.e. neurotmesis in Seddon’s classification^30^ or ≥ 3rd degree nerve injury in Sunderland’s classification^31^, prevents complete recovery. On the other hand, damage to the axon and myelin sheath without endo-, peri- or epineurium damage, i.e., axonotmesis in Seddon’s classification and 2nd degree nerve injury in Sunderland’s, leads to complete recovery. Conceivably ITST in the present study may limit the severity of facial nerve damage and prevent the damage to the endo-, peri- and epineurium that leads to subsequent incomplete recovery.

This effect might reduce the number of patients who present the most aggravated facial nerve degeneration in the concurrent ITST group. For example, the number of cases exhibiting aggravated (≥ 95%) degeneration^8^ in the early phase (≤ 10 days after onset), who experience low recovery rates is much smaller in the concurrent ITST group ([2/8, 25%] compared to the control group [9/21, 42%]). Considering that the concurrent ITST and control groups had similar baseline characteristics in terms of age, time after onset of first steroid therapy, and most severe grade, this finding appears to result from the extra neuroprotective effect of ITST, which reduces damage caused by neuroinflammation^27^, leading to relatively milder degeneration as a result.

In the previous study, we demonstrated a significant suppression of the hypothalamic-pituitary axis with ITST compared to the control group, despite the steroid administered having lower glucocorticoid activity, which strongly suggests dexamethasone transfer to the facial canal when administered trans-tympanically^22^. The greater benefit of concurrent ITST in the present study may be ascribed to the neuroprotective effect of the high steroid concentration delivered by ITST. Steroids are more efficacious at high doses than at low doses. For example, when a prednisolone dose ≥ 450 mg is administered, the relative risk of an unsatisfactory recovery decreases significantly from 0.96 to 0.56^32^. Furthermore, a recent meta-analysis concluded that the recovery rate is higher in patients who receive an initial daily prednisolone dose of ≥ 100 mg, than in those who receive standard-of-care with an initial dose of 50–60 mg (an odds ratio of 0.42)^33^. These consistent conclusions strongly support the efficacy of high-dose steroid therapy on facial recovery in Bell’s palsy. Interestingly, this is also the case in Ramsay Hunt syndrome, in which high-dose steroid therapy is similarly effective in facial recovery in patients with poor ENoG test results^34,35^.

In the current management of Bell’s palsy, surgical decompression is the most common treatment option when a poor prognosis is anticipated by electrodiagnosis^36,37^. A recent meta-analysis concluded that this surgery is an effective supplemental treatment in patients with an ENoG value of ≤ 10%, when it is performed within 2 weeks after onset^38^. However, regardless of accumulated evidence, this technique is currently not routinely performed, it is reported that only 4.7% of otologists perform this procedure more than once a year^39^, due to the technical difficulties associated with the procedure and the limited data supporting its effectiveness.

Compared to surgical decompression, the intratympanic steroid injection is more widely practiced worldwide. It is already an established treatment for sudden sensorineural hearing loss^40^. It is also minimally invasive compared to facial nerve decompression surgery. The procedure can be performed simply by perforating the tympanic membrane with a fine needle, such as a 23G needle (0.643 mm, outer diameter). Moreover, costs and risks are lower than with surgical interventions.

The appropriate timing for initiating ITST has not been studied so far. It is likely to be most effective if initiated within 72 h after onset, as is the case for systemic steroids^37^. However, although a patient’s facial palsy will reach its worst grade by 72 h^37^ post onset, a prognosis with electroneurography is only possible after 72 h, and usually reaches its worst level in the second week of onset^8^. A high HB grade for facial paralysis at 72 h does not necessarily indicate a poor prognosis, which will be determined using ENoG after day 7 or later^37^. This creates a challenge for the clinician because ITST needs to be started early in patients with a poor prognosis to assist their recovery, but these patients who most need early intervention cannot be reliably identified at the time the treatment needs to start. Currently, no other technique offers as accurate a prognostic prediction as does timely ENoG. This is one disadvantage of ITST against surgical decompression, which can be performed as late as day 14 after onset after carefully selecting candidates by electrodiagnosis^39^. A different approach is needed to accurately evaluate the severity of intratemporal Wallerian degeneration. Early diagnosis by transcranial magnetic stimulation^41^ or imaging^42^ may be directions to explore, but further work will be needed to establish the techniques^9^.

The limitations of this study include small sample sizes due to the scarcity of patients^13,17^, its nonrandomized design, lack of blinding, and the use of historical controls. In addition, the patient enrollment based on ENoG testing performed after starting interventions may have caused a selection bias. Nevertheless, the efficacy of ITST observed in the present study improves facial outcomes in patients in whom a poor prognosis is anticipated, implying that intratympanic injection of steroids may help to overcome the remaining shortcomings of current Bell’s palsy management.

In conclusion, Bell’s palsy that presents with an ENoG value ≤ 10% within 3 weeks after onset of facial palsy is correlated with a poor prognosis when treated only with systemic steroids. In this study, we showed that concurrent ITST significantly improved the recovery rate in this subgroup from 43 to 88%. This observation suggests that ITST has the potential to benefit patients with Bell’s palsy and severe nerve degeneration in whom a poor prognosis is anticipated by electrodiagnostic testing.

## Methods

### Ethical standards

The study protocol was approved by the Institutional Review Board of Nagoya City University (registration number, 41-13-0004, UMIN trial registration: umin.ac.jp; UMIN000031107). All procedures performed in studies involving human participants were in accordance with the ethical standards of the institutional and national research committee and with the 1964 Helsinki declaration and its later amendments or comparable ethical standards. Patients in the control group were informed about the study and given the opportunity to opt out of participating. The study protocol was approved by the Institutional Review Board of Nagoya City University, with a waiver of informed consent for retrospective medical records review (approval number, H27-845).

### Participants and study design

In this study, we selected participants for whom electrodiagnostic testing indicated a poor prognosis in the previous study, an open-label trial which included a historical control group that investigated the efficacy of early intervention ITST concurrently with standard therapy^22^. Detailed descriptions of eligibility criteria and recruitment methods have been published previously. The primary outcome measure was restoration of a House-Brackmann (HB) grade of I^43^, and the secondary outcome measure was the extent of recovery of facial nerve function, as assessed by the HB grade recorded 1, 3, 6, 9, and 12 months after the onset of facial palsy.

The subjects in the concurrent ITST group (ITST group) were recruited from the otolaryngology clinic at Nagoya City University Hospital, a tertiary referral center in Nagoya, Japan, between March 2014 and December 2015 and those in the historical control group (control group) between January 2007 and December 2015^22^. The study protocol was approved by the Institutional Review Board of Nagoya City University (registration number, 41-13-0004, UMIN trial registration: umin.ac.jp; UMIN000031107, registered on February 1, 2018, retrospectively registered). Patients in the control group were informed about the study and given the opportunity to opt out of participating. The study protocol was approved by the Institutional Review Board of Nagoya City University, with a waiver of informed consent for retrospective medical records review (approval number, H27-845).

Patients were eligible for enrollment if they were ≥ 20 years of age and had a diagnosis of Bell’s palsy, i.e., acute unilateral facial nerve paralysis of unknown cause^37^ and were able to initiate the protocol within 7 days of the onset of paralysis. ITST was administered for 10 consecutive days using a previously reported regimen^22^, concurrent with systemic prednisolone (tapered from 60 mg/day, total 410 mg) and an appropriate dose of valaciclovir^5^ starting on the day of enrollment (Table 1). Prognosis was assessed electrophysiologically with electroneurography (ENoG). ENoG was evaluated within the prescribed time frame for predicting recovery, 3 to 21 days after onset of facial palsy^9^. We identified patients in both the ITST and control groups as having a poor prognosis (≥ 90% degeneration [≤ 10% of the ENoG value] of the facial nerve on ENoG) within this time frame. The sample size was not set for hypothesis testing since the present study was an exploratory analysis.

### Patient evaluation

The severity of facial nerve dysfunction in both study groups was assessed by facial nerve specialists working independently, using the HB grading system (I, normal; VI, total facial nerve paralysis)^43^ and the Yanagihara grading system (40, normal; 0, total facial nerve paralysis)^44^. Electroneurography was performed using an MEB-2300-Neuropack X1 system (Tokyo, Japan).

### Statistical methods

All data are presented as the mean ± standard error of the mean unless otherwise stated. Differences between the concurrent ITST and historical control groups were assessed for statistical significance using Student’s *t*-test. Nonparametric analysis was performed for data that were not distributed normally using the Mann–Whitney rank sum test (SigmaPlot, Systat Inc, San Jose, CA). Fisher’s exact test and the chi-square test were used to analyze the statistical significance of differences in recovery rates (SigmaPlot). Further details concerning data collection and statistical analyses are provided in the Supplemental Methods.

## Supplementary Information

Supplementary Information.
