# Supplementary material for: Intratympanic steroid therapy for Bell’s palsy with poor prognostic results
Source: Sci Rep. 2021 Apr 13;11:8058. doi: 10.1038/s41598-021-87551-x (PMC8044212; doi:10.1038/s41598-021-87551-x)
Supplement: Supplementary file 1 — Supplementary Information. [file 41598_2021_87551_MOESM1_ESM.docx]

Scientific Reports

**Supplementary Information**

**Intratympanic steroid therapy for Bell’s palsy with poor prognostic results**

Akira Inagaki, Sachiyo Katsumi, Shinji Sekiya, Shingo Murakami

**Supplementary Methods**

**Supplementary Figures**

**Supplementary Fig 1.** Flow chart showing the process used to enroll patients in the study.

**Supplementary Fig 2.** Additional analysis of the efficacy of concurrent ITST in patients with Bell’s palsy with 95% or more facial nerve denervation on electroneurography using the Yanagihara grading system.

**Supplementary Fig 3.** Relationship between the denervation on electroneurography and facial nerve recovery using the Yanagihara grading system.

**Supplementary Tables**

**Supplementary Table 1**. Eligibility criteria.

**Supplementary Table 2.** Recovery of facial nerve function in patients with Bell’s palsy according to House–Brackmann grade.

**Supplementary Methods**

***Participants and setting***

During the concurrent ITST procedure, patients were instructed to remain supine with the head tilted 40°–45° to the nonaffected side and bent slightly backwards. After administration of local anesthesia, we introduced a small ventilation hole into the tympanic membrane and injected 0.5 ml of dexamethasone (3.3 mg/ml) into the tympanic cavity using a 23-G spinal needle as previously reported [1]. Patients were asked to avoid head movement, swallowing, and yawning for 15 min after the injection.

***Patient Evaluation***

At least two assessments were performed 4–7 days apart at some time in the 5–17 days after onset of facial palsy; the mean value was recorded. Recovery was evaluated at 1 month (days 25–35), 3 months (days 75–105), 6 months (days 167–197), 9 months (days 258–288), and 12 months (days 350–380).

***Statistical methods***

Patients who had zoster without rash, who were receiving other treatments that could affect the facial nerve outcome (e.g., facial nerve decompression surgery), or who were confirmed not to have Bell’s palsy during the 12-month follow-up period after onset of symptoms were excluded. Patients were diagnosed as positive for zoster without rash (zoster sine herpete) when a ≥8-fold elevation of the anti-VZV titer was detected on complement-fixation tests [2].

**References**

1. Inagaki A, Minakata T, Katsumi S, Murakami S (2019) **Concurrent Treatment With Intratympanic Dexamethasone for Moderate-Severe Through Severe Bell's Palsy**, *Otology & neurotology: official publication of the American Otological Society, American Neurotology Society [and] European Academy of Otology and Neurotology*.

2. Murakami S, Honda N, Mizobuchi M, Nakashiro Y, Hato N, Gyo K (1998) **Rapid diagnosis of varicella zoster virus infection in acute facial palsy**, *Neurology*, **51**(4):1202-1205.


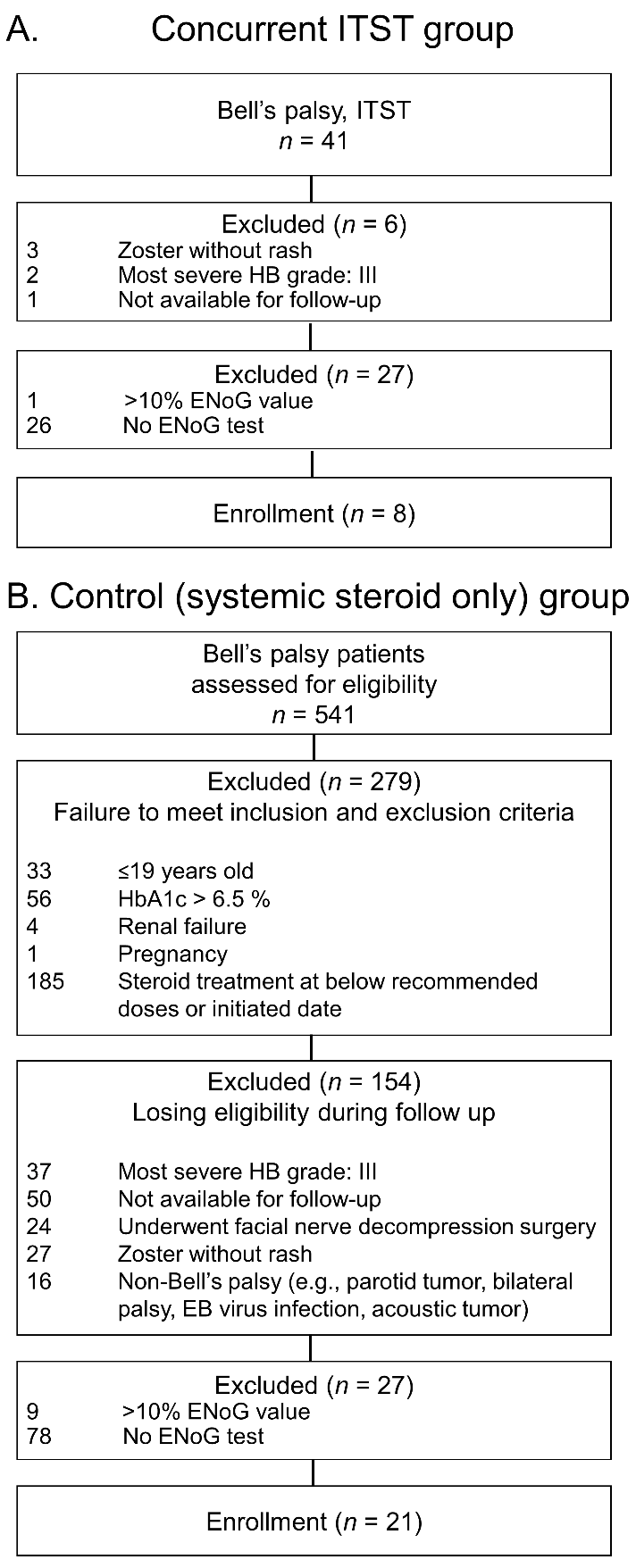
**Supplementary Figures**

**Supplementary Fig 1**. Flow chart showing the process used to enroll patients in the study. (A) Concurrent ITST group, (B) systemic steroid group. ENoG, electroneurography; HB, House-Brackmann; HbA_1c_, glycated hemoglobin; ITST, intratympanic steroid therapy

**
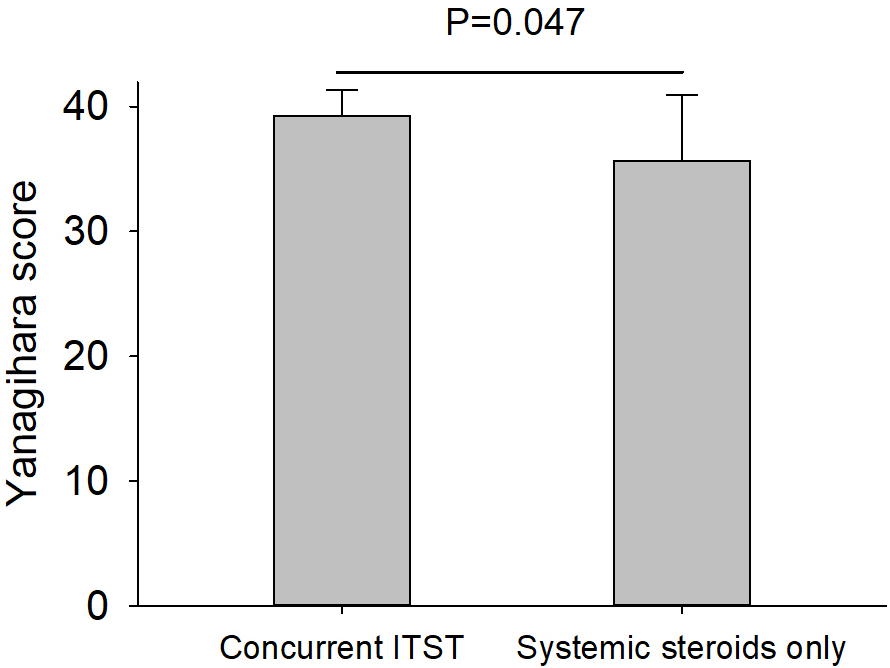
**

**Supplementary Fig 2.** Efficacy of concurrent ITST in patients with Bell’s palsy with 95% or more facial nerve denervation on electroneurography using the Yanagihara grading system.


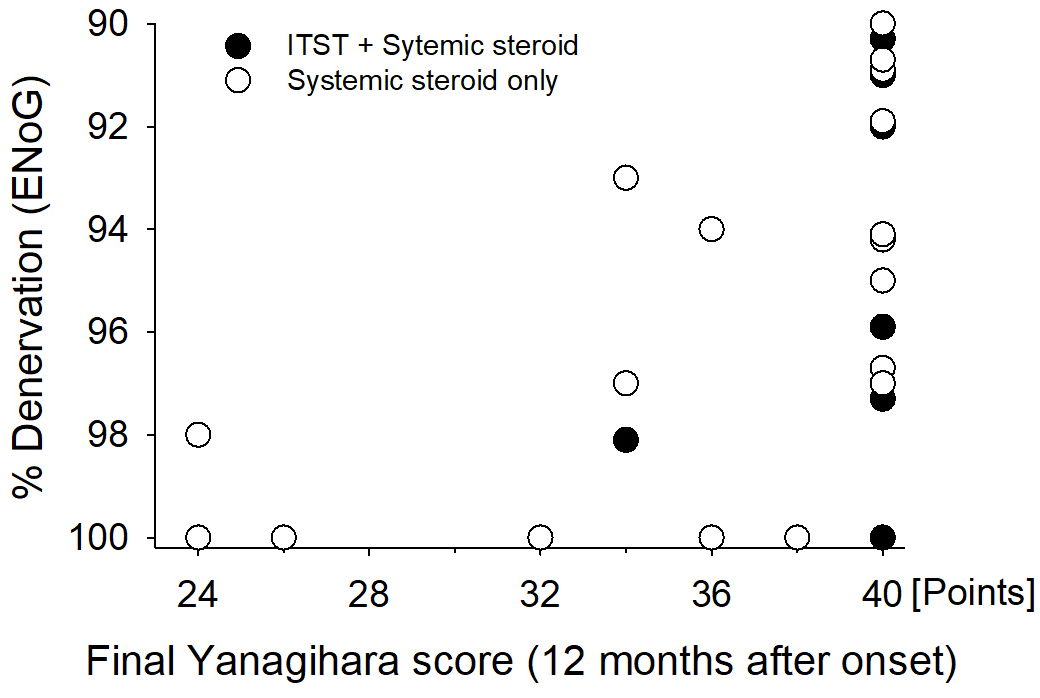


**Supplementary Fig 3.** Relationship between the denervation on electroneurography and facial nerve recovery using the Yanagihara grading system.

**Supplementary Tables**

**Supplementary Table 1.**  Eligibility criteria

Inclusion criteria

1. Diagnosis with Bell’s palsy

2. Potential to initiate protocol within 7 days of onset

3. Twenty years of age or older

4. Most severe facial palsy (House-Brackmann grade IV or higher) for control patients

5. Demonstration of an electroneurography value ≤10% within 3 weeks after onset of facial palsy.

Exclusion criteria

1. Presence of nonviral inflammation in the middle ear

2. Presence of bacterial disease at carriage state.

3. Presence of middle ear or inner ear anomaly that would make injection into middle ear impossible.

4. Glycated hemoglobin >6.5%.

5. Blood urea nitrogen >25 mg/dl or serum creatinine level >2.0 mg/dl.

6. Alanine transaminase >100 U/l or aspartate transaminase >100 U/l.

7. Severely protracted wound healing.

8. Signs of central facial palsy or other neurological disease that could potentially affect facial function

9. Pregnancy or possible pregnancy

10. Participation in other clinical trials within 3 months

11. Recurrent facial palsy

12. History of facial nerve decompression surgery

13. Serological diagnosis of zoster sine herpete (for Bell’s palsy)

14. Pretreatment other than 60 mg of prednisolone in combination with 1000 mg oral valaciclovir within the previous 2 days (for control patients). Treatment with prednisolone and/or valaciclovir at doses lower than those in Table 1 (in patients receiving concurrent intratympanic steroid therapy).

15. Inappropriate for participation in the trial in the opinion of the investigators.

**Supplementary Table 2.** Recovery of facial nerve function in patients with Bell’s palsy according to House–Brackmann grade

|  | Concurrent ITST/control | Difference/*P*-value |
| --- | --- | --- |
| Worst | 5.38 ± 0.18/  5.19 ± 0.11 | -0.19/0.415 |
| 1 month | 4.50 ± 0.27/  4.43 ± 0.15 | -0.07/0.742 |
| 3 months | 3.13 ± 0.30/  3.43 ±0.24 | 0.30/0.432 |
| 6 months | 1.75 ± 0.37/  2.38 ± 0.23 | 0.63/0.111 |
| 9 months | 1.50 ± 0.27/  2.24 ± 0.28 | 0.74/0.109 |
| 12 months | 1.13 ± 0.13/  1.71 ± 0.16 | 0.59/0.035* |

**P*<0.05
